# PARP inhibitors in ovarian cancer: Sensitivity prediction and resistance mechanisms

**DOI:** 10.1111/jcmm.14133

**Published:** 2019-01-22

**Authors:** Xuan Jiang, Xiaoying Li, Weihua Li, Huimin Bai, Zhenyu Zhang

**Affiliations:** ^1^ Department of Obstetrics and Gynecology, Beijing Chao‐yang Hospital Capital Medical University Beijing China

**Keywords:** BRCA1/2, homologous recombination deficiency, ovarian cancer, PARP inhibitor, resistance mechanism

## Abstract

Poly (ADP‐ribose) polymerase (PARP) inhibitors have provided great clinical benefits to ovarian cancer patients. To date, three PARP inhibitors, namely, olaparib, rucaparib and niraparib have been approved for the treatment of ovarian cancer in the United States. Homologous recombination deficiency (HRD) and platinum sensitivity are prospective biomarkers for predicting the response to PARP inhibitors in ovarian cancers. Preclinical data have focused on identifying the gene aberrations that might generate HRD and induce sensitivity to PARP inhibitors in vitro in cancer cell lines or in vivo in patient‐derived xenografts. Clinical trials have focused on genomic scar analysis to identify biomarkers for predicting the response to PARP inhibitors. Additionally, researchers have aimed to investigate mechanisms of resistance to PARP inhibitors and strategies to overcome this resistance. Combining PARP inhibitors with HR pathway inhibitors to extend the utility of PARP inhibitors to BRCA‐proficient tumours is increasingly foreseeable. Identifying the population of patients with the greatest potential benefit from PARP inhibitor therapy and the circumstances under which patients are no longer suited for PARP inhibitor therapy are important. Further studies are required in order to propose better strategies for overcoming resistance to PARP inhibitor therapy in ovarian cancers.

## 1 INTRODUCTION

Epithelial ovarian cancer is the most lethal gynecologic malignancy, and most patients present with advanced‐stage disease.^1^ After standard primary treatment, most patients develop recurrence with a median progression‐free survival (PFS) time of 18 months; furthermore, treatment efficacy diminishes over time.^2^ Hence, new treatment methods are warranted to improve the prognosis of ovarian cancer patients.

Poly (ADP‐ribose) polymerase (PARP) inhibitors are oral small‐molecule inhibitors of PARP enzymes 1, 2 and 3, which play a critical role in the repair of DNA single‐strand breaks (SSBs) via the base excision repair (BER) pathway. Breast Cancer Susceptibility Gene (BRCA)1 and BRCA2 are two key tumour suppressors in the repair of DNA double‐strand breaks (DSBs) via the homologous recombination (HR) repair pathway.^3^ PARP inhibition in BRCA mutant tumour cells could induce “synthetic lethality”, which occurs from the simultaneous targeting of two DNA repair pathways and results in profound cytotoxicity to tumour cells while sparing normal cells.^4^, ^5^ PARP inhibitors are the first Food and Drug Administration (FDA)‐approved biological agent for ovarian cancer based on personalized features of the patient's cancer.^6^ Now in the Europe, olaparib is approved as maintenance treatment in platinum‐sensitive, relapsed, BRCA1/2 mutated ovarian cancer after a complete or partial response (CR/PR) to platinum‐based chemotherapy; niraparib is approved as maintenance treatment in platinum‐sensitive, relapsed, ovarian cancer after a CR/PR to platinum‐based chemotherapy; rucaparib is approved as third‐line treatment in BRCA1/2 mutated, platinum sensitive, relapsed high‐grade ovarian cancer. In the United States, olaparib is approved as fourth‐line treatment in BRCA1/2 mutated, advanced ovarian cancer and first‐line maintenance treatment in BRCA‐mutated, advanced ovarian cancer after a CR/PR to first‐line platinum‐based chemotherapy; rucaparib is approved as third‐line treatment in BRCA1/2 mutated, advanced ovarian cancer; niraparib is approved as maintenance treatment in platinum‐sensitive, recurrent ovarian cancer after a CR/PR to platinum‐based chemotherapy. There are some minor differences between the indications of these drugs in Europe and USA, based on different clinical trials.^7^, ^8^, ^10^ All three PARP inhibitors approved for ovarian cancer treatment have substantial PFS advantages over placebo in the maintenance setting.^11^, ^12^ Clinical trials of these three PARP inhibitors are ongoing, and these drugs are expected to have substantial PFS advantages over placebo in the treatment setting as well.

BRCA1/2 mutations remain the strongest genetic indicators of sensitivity to PARP inhibitors.^13^ However, 40 − 70% of BRCA1/2‐mutated ovarian cancers fail to respond to PARP inhibitors.^14^, ^15^, ^16^, ^17^ In addition, the remarkable efficacy of PARP inhibitors in ovarian cancer is not restricted to patients with germline BRCA1/2 mutations but extends to those with tumours with HR repair pathway deficiencies.^18^ Tumours with mutations in certain genes that are not directly involved in DNA repair but are related to the HR pathway or influence the effect of HR pathway genes are sensitive to PARP inhibitors.^19^ This review discusses preclinical and clinical data that describe methods of predicting the response to PARP inhibitors, the potential mechanisms of resistance to PARP inhibitors, and measures to circumvent resistance to PARP inhibitors.

## 2 BIOMARKERS PREDICTING CLINICAL BENEFIT FROM PARP INHIBITORS

### 2.1 High correlation between platinum sensitivity and PARP inhibitor response

"BRCAness" is used to describe the phenotype shared between non‐BRCA1/2‐mutated ovarian cancers and BRCA1/2‐mutated ovarian cancers.^20^ The molecular characteristics of "BRCAness" might lie in the aberration of certain genes involved in the HR repair pathway, such as BRCA1, BRCA2, ATM, BARD1, BRIP1, CHEK1, CHEK2, FAM175A, MRE11A, NBN, PALB2, RAD51C and RAD51D.^21^ Ovarian cancers with a "BRCAness" phenotype exhibit high sensitivity to both platinum and PARP inhibitors, and the overall survival rate is higher in these cancers than in ovarian cancers without a “BRCAness” phenotype.^21^, ^22^ Hence, platinum sensitivity might indicate the molecular characteristics of BRCAness and be a potential phenotypic marker for PARP inhibitor sensitivity.

The PARP inhibitor response is closely related to platinum sensitivity in clinical trials of PARP inhibitors. Arms in clinical trials of PARP inhibitors were balanced for platinum sensitivity, prior chemotherapeutic regimen and prior lines of chemotherapy.^23^ Olaparib is the earliest and most extensively investigated PARP inhibitor, and the relationship between the olaparib response and platinum sensitivity has been deeply evaluated. A significant association between the olaparib clinical benefit rate and platinum sensitivity in BRCA1/2‐mutated ovarian cancers was confirmed; the clinical benefit rate of olaparib therapy was 69.2% in platinum‐sensitive, 45.8% in platinum‐resistant and 23.1% in platinum‐refractory BRCA1/2‐mutated ovarian cancers.^16^ The response to olaparib is best in germline BRCA1/2‐mutated, platinum‐sensitive ovarian cancers and worst in BRCA1/2 wild‐type, platinum‐resistant ovarian cancers.^14^, ^15^, ^24^, ^25^ Thus, platinum sensitivity may be a good predictor of the PARP inhibitor response in BRCA1/2 wild‐type ovarian cancers; indeed, in a phase II trial of olaparib, half of the platinum‐sensitive ovarian cancers with wild‐type BRCA1/2 responded to olaparib, but only 4% of the platinum‐resistant ovarian cancers with wild‐type BRCA1/2 responded. However, platinum resistance might not be sufficiently powerful to rule out clinical benefit from PARP inhibitors in BRCA‐mutated cancers, since 60% of platinum‐sensitive, BRCA1/2‐mutated ovarian cancers and 33% of platinum‐resistant, BRCA1/2‐mutated ovarian cancers in the same trial responded to olaparib.^15^

Notably, a response to platinum does not guarantee a response to PARP inhibitors. Unlike PARP inhibitor sensitivity, platinum sensitivity may result from defective nucleotide excision repair (NER).^26^ The platinum‐induced interstrand and intrastrand cross‐links (ICLs) between purine bases in DNA are extremely deleterious and are more cytotoxic than the SSBs caused by PARP inhibitors.^27^, ^28^, ^29^, ^30^ In addition, the partial restoration of HR by genetic alterations such as 53BP1 loss in BRCA1‐mutated tumour cells (discussed below) is insufficient to repair the complex cross‐links caused by platinum agents; tumours with partially restored HR repair retain sensitivity to platinum but exhibit resistance to PARP inhibitors.^29^, ^31^, ^32^ Olaparib‐resistant, heavily pretreated BRCA1/2‐mutated ovarian cancers have been observed to retain the potential to respond to subsequent chemotherapy, even platinum‐based agents. In addition, an increased platinum‐to‐platinum interval during olaparib treatment indicated a response to subsequent platinum agents.^33^ However, an increased number of previous platinum‐based therapies may indicate the development of secondary mutations that restored the functions of the BRCA1/2 or RAD51C/D genes and influenced the sensitivity to PARP inhibitors.^23^

### 2.2 Potentially relevant gene aberrations and combination strategies for PARP inhibitor sensitization

To identify which gene aberrations might induce synthetic lethality with PARP inhibitors, researchers studied the five major DNA damage repair pathways operational in cells.^3^, ^34^ The combination of NER deficiency and PARP inhibition has been demonstrated not to be synthetically lethal.^26^ However, although NER deficiency does not lead to synthetic lethality, the combination of PARP inhibition and deficiencies in other DNA repair pathways such as BER, HR, non‐homologous end joining (NHEJ) and even mismatch repair (MMR) has been demonstrated to be synthetically lethal.^35^ Since the HR pathway is a high‐fidelity repair pathway for accurately repairing DSBs and involves the BRCA1/2 proteins, researchers have focused on the HR pathway.

**Figure 1:**
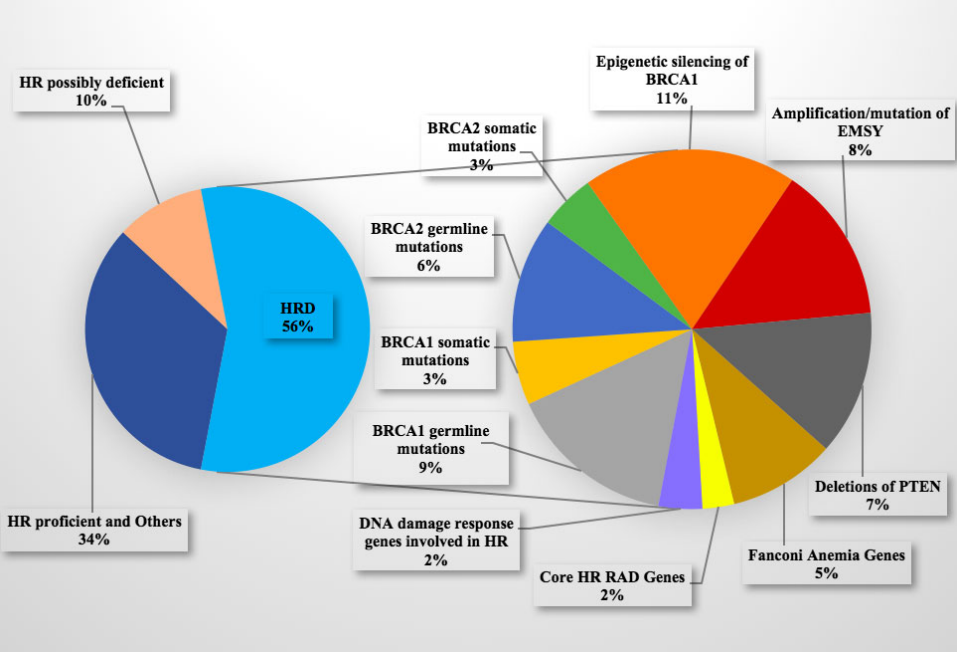
According to The Cancer Genome Atlas (TCGA), ovarian cancers can be classified as HR‐proficient and other (34%), possibly HR‐deficient (10%) (including those with alterations in 42 other potentially relevant HR genes, such as BLM, ERCC4, TP53BP1, RPA1 and XRCC3) and HR‐deficient (56%) (including those with alterations of BRCA1/2; the amplification or mutation of EMSY; the deletion of PTEN, Fanconi anemia genes [including FANCA, FANCB, FANCC, FANCD2, FANCE, FANCF, FANCG, FANCI, FANCL, FANCM, PALB2 and C19orf40], core HR RAD genes [including RAD50, RAD51, RAD51C, RAD51L1, RAD51L3, RAD52, RAD54B and RAD54L] or HR‐related DNA damage response genes [including ATM, ATR, CHEK1 and CHEK2])^27^, ^36^

**Figure 2:**
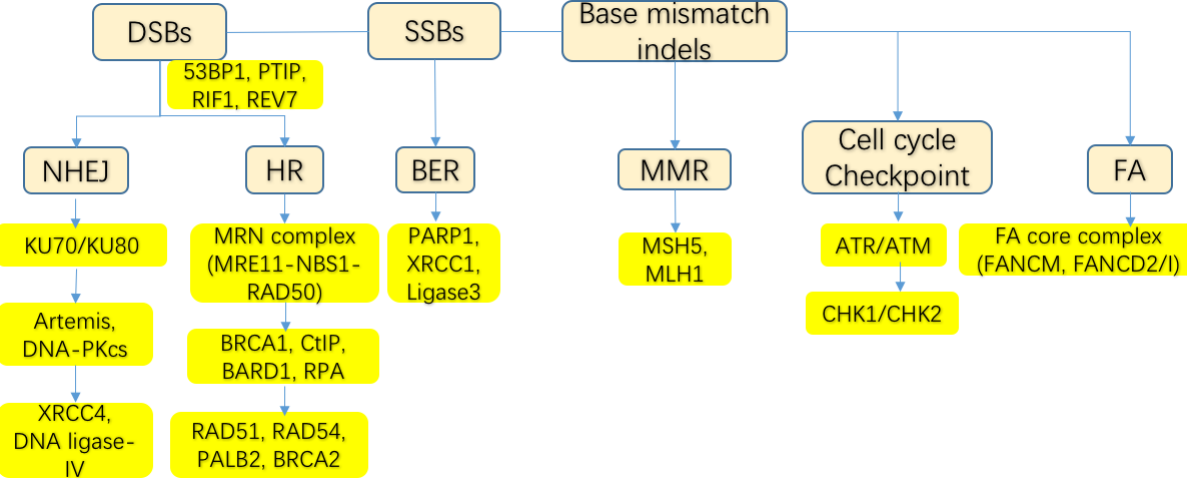
The molecular process of DNA damage repair associated with PARP inhibitor sensitivity and resistance. 1) During the repair of DSBs by NHEJ, damage is recognized and bound by Ku70‐Ku80 heterodimers, DNA‐PKcs and Artemis are activated, and XRCC4 and DNA ligase‐IV are recruited to complete DNA end joining. NHEJ occurs throughout the cell cycle and directly ligates the ends of a DSB. The loss of the abovementioned crucial proteins in the NHEJ pathway might induce resistance to PARP inhibitors. 2) During the repair of DSBs by HR, DNA lesions are recognized by the MRE11‐NBS1‐RAD50 (MRN) complex, and DNA end resection is initiated. In a PALB2‐dependent fashion, BRCA2 is recruited, which loads RAD51 onto the DNA to mediate strand invasion on the homologous sister chromatid. HR occurs mainly in the S and G2 phases. The switch between HR and NHEJ depends on the activity of S phase CDKs, which phosphorylate CtIP in order to activate the MRN complex and stimulate DNA end resection, which is regulated by 53BP1, REV7 and RIF1. The abovementioned crucial proteins in the HR pathway might thus affect PARP inhibitor sensitivity. 3) In addition, the cell cycle checkpoint pathway and the FA‐BRCA1 pathway are involved in HR, and crucial proteins in these pathways are associated with PARP inhibitor sensitivity^27^, ^46^

Approximately 50% of high‐grade serous ovarian cancers (HGSOCs) are deficient in HR because of germline or somatic mutations in BRCA1/2 (20%), the epigenetic silencing of BRCA1 (11%), the amplification or mutation of EMSY (8%), the deletion of PTEN (7%), the hypermethylation of RAD51C (3%), mutations in ATM or ATR (2%) or mutations in Fanconi anemia genes (5%).^36^ The key genes and modulators of the HR pathway that might induce synthetic lethality with PARP inhibitors are under investigation. Figure 1 shows the aberrations in HR genes or potentially relevant HR genes and Figure 2 presents the molecular process of DNA damage repair associated with PARP inhibitor sensitivity and resistance.

To identify tumours with a deficiency in HR genes, targeted capture and the massively parallel genomic sequencing of multiple‐gene panels would be required. A 60‐gene panel, referred to as the BRCAness profile, was developed to distinguish BRCA‐like tumours from non‐BRCA‐like tumours. The BRCAness profile accurately predicted platinum sensitivity in 8 of 10 ovarian tumours and accurately identified PARP inhibitor sensitivity and resistance in all four cancer cell lines tested.^37^ A 30‐gene panel was developed to target all known breast and ovarian cancer genes, including BRCA1 and BRCA2 as well as 11 HR‐related genes.^21^ A 21‐gene panel was developed to target germline mutations in tumour suppressor genes, including BRCA1, BRCA2, and other genes known to cause inherited breast or ovarian carcinoma.^38^ These multiple‐gene panels are prospective for identifying tumours that are sensitive to PARP inhibitors.

Germline or somatic BRCA1/2 mutations have been demonstrated to significantly affect tumour sensitivity to PARP inhibitors.^39^ The epigenetic silencing of BRCA1 represented 15% of sporadic ovarian cancers in a population‐based study. In addition, the epigenetic silencing of BRCA1 was demonstrated to be excluded in hereditary ovarian cancers, and cancers with epigenetic BRCA1 silencing did not exhibit BRCA1 protein expression by immunohistochemistry.^40^ Furthermore, BRCA1 hypermethylation conferred the same degree of sensitivity to PARP inhibitors as did BRCA1/2 mutations in a breast cancer cell line. In addition, the growth of xenograft tumours with epigenetically silenced BRCA1 was reduced by PARP inhibitor treatment.^41^, ^42^ However, in a panel of 39 ovarian cancer cell lines representing all histologic subtypes, as well as in a panel of 21 breast cancer cell lines, methylation of the BRCA1 promoter was not detected.^35^, ^43^ Furthermore, according to The Cancer Genome Atlas (TCGA), unlike BRCA1/2 mutations, ovarian cancers with epigenetic silencing of BRCA1 through promoter hypermethylation appear not to respond as favourably to platinum and not to exhibit improved survival. This raises the question whether patients with epigenetic silencing of BRCA1 may respond more favourably to PARP inhibitors.^36^

The DNA recombinase RAD51 is a crucial downstream protein in the HR repair pathway.^44^ RAD51 is relocalized within the nucleus in response to DNA damage to form distinct foci, which are thought to represent assemblies of proteins at these sites of HR repair. PARP inhibition leads to the failure of SSB repair via the BER pathway, and persistent SSBs result in stalled replication forks and subsequently develop into DSBs. In HR‐proficient cells, these DSBs can be repaired by error‐free HR accompanied by an increase in RAD51 foci formation, whereas in HR‐deficient cells, repair by error‐prone NHEJ results in genomic instability, chromosomal aberrations, cell cycle arrest and ultimately cell death.^45^ Hence, RAD51 nuclear foci can be a surrogate biomarker for HR repair functionality and can be detected by a functional RAD51 immunofluorescence assay. The absence of RAD51 foci formation represents a functional deficiency of HR repair.^46^ The RAD51 foci formation assay successfully predicted the in vitro response to PARP inhibitors in primary cultures from the ascitic fluid of ovarian cancer patients.^22^, ^45^ Using RAD51 foci as a biomarker, PARP inhibitor cytotoxicity was observed in 93% of HR‐deficient tumours but in no HR‐proficient tumours.^45^ Furthermore, irradiation‐induced RAD51 foci formation assay accurately predicted which patient‐derived xenograft (PDX) models were sensitive to PARP inhibition in vitro and in vivo.^47^ However, in a study by AlHilli et al, the decreased formation of RAD51 foci failed to predict a response to PARP inhibitors in HGSOC PDX models.^19^

Five RAD51 paralogs exist: RAD51B, RAD51C, RAD51D, XRCC2 and XRCC3. Germline mutations in both RAD51C and RAD51D were identified in families with ovarian cancer. Loss‐of‐function mutations in RAD51D predispose individuals to ovarian cancer but not to breast cancer.^44^ Germline RAD51D mutations were found in 0.8% of subjects with ovarian cancer not selected for a family history of ovarian cancer.^48^ When treated with RNA interference targeting RAD51D, tumour cells exhibit a sensitivity to olaparib similar to that achieved by the silencing of BRCA2.^44^ RAD51C acts sequentially with RAD51 at the DNA damage site to repair DNA damage, and RAD51C depletion leads to impaired RAD51 foci formation, resulting in impaired DNA repair. RAD51C promoter methylation has been studied thoroughly. RAD51C was found to be densely methylated in both cancer cell lines and tumour tissue samples, resulting in low RAD51C expression. RAD51C‐deficient cancer cells were more sensitive to olaparib than were RAD51C‐proficient cancer cells, and the silencing of RAD51C in olaparib‐resistant cell lines could enhance olaparib sensitivity. Olaparib significantly suppressed RAD51C‐deficient tumour growth in a xenograft model.^49^ In addition, RAD51C promoter methylation was demonstrated to be associated with PARP inhibitor response in a PDX model.^19^

BCCIP is an important cofactor for BRCA2 in tumour suppression. BRCC is a holoenzyme complex containing BRCA1, BRCA2, RAD51, BRCC36 and BRCC45. EMSY binds BRCA2 within exon 3 of BRCA2 and is capable of silencing the activation potential of this exon. The protein kinase Aurora‐A (AURKA) inhibits RAD51 recruitment to DNA DSBs, decreases DSB repair by HR and sensitizes cancer cells to PARP inhibitors. Hence, the low expression of BCCIP or BRCC3 and the amplification of EMSY or AURKA were demonstrated to be significantly associated with in vitro PARP inhibitor sensitivity in a series of 39 ovarian cancer cell lines.^35^ Whether PTEN deficiency causes HR deficiency (HRD) in the repair of DNA DSBs remains controversial.^50^, ^51^, ^52^ PTEN deficiency might alter multiple cell cycle checkpoints, possibly decreasing the time available for DNA damage repair.^52^ PTEN deficiency has been observed to sensitize cancer cells to PARP inhibitors both in vitro and in vivo.^50^, ^51^ However, in the aforementioned 39 ovarian cancer cell lines, PTEN mutations were not significantly associated with in vitro sensitivity to PARP inhibitors.^35^

ATR and its downstream effector CHK1 are two important kinases in the replication checkpoint signaling pathway. The ATR‐CHK1 pathway can be activated by DNA damage and replication stress; the activity of this pathway arrests cell cycle progression and helps cells survive these genotoxic stresses. In addition, this pathway stabilizes replication forks and prevents the collapse of replication forks into DNA DSBs. The inactivation of ATR/CHK1 results in substantial DSBs, the loss of the G2‐M checkpoint, and the premature entry of cells with high‐level DSBs into mitosis, resulting in chromosome aberrations and cell apoptosis. Furthermore, ATR participates in the HR pathway by phosphorylating and regulating BRCA1 and other important proteins.^53^, ^54^ The depletion of ATR but not CHK1 using siRNAs or small molecular inhibitors has been demonstrated to sensitize ovarian cancer cells (including HR‐proficient cells) to PARP inhibitors. In addition, ATR inhibition disrupted the function of HR repair and further sensitized cells with HRD to PARP inhibition.^53^ Combined treatment with a PARP inhibitor and an ATR/CHK1 inhibitor decreased the viability and colony‐forming ability of both BRCA mutant and HR‐proficient cells. The PARP‐ATR inhibitor combination caused the complete regression of BRCA‐mutated ovarian cancer PDX tumours, while the PARP‐CHK1 inhibitor combination led only to tumour suppression rather than to tumour regression. Nevertheless, compared with the PARP inhibitor alone, both of the combinations showed significantly improved efficacy.^54^ The phase II trial combining an ATR inhibitor (AZD6738) with a PARP inhibitor (olaparib) to treat recurrent ovarian cancers is ongoing (CAPRI: NCT 03462342).

ATM and CHK2 are also important cell cycle checkpoint proteins.^55^ ATM phosphorylates CHK2 and promotes CHK2 activation. In turn, CHK2 rapidly hyperphosphorylates BRCA1 at multiple sites, switching error‐prone NHEJ to error‐free HR^56^, ^57^ CHK2 also helps BRCA1 control NHEJ and reduces the mutagenic potential of NHEJ.^58^ ATM deficiency induces both DNA DSB repair defects and cell cycle checkpoint deficiencies. Checkpoint signals resulting from the detection of DNA damage cannot induce cell cycle arrest, thus preventing the opportunity for cellular repair systems to respond and resulting in cell apoptosis. Hence, ATM defects impair HR‐mediated DSB repair.^59^ Low ATM expression was demonstrated to be significantly associated with PARP inhibitor sensitivity both in vitro and in vivo.^35^, ^60^ Consistent with the above findings, a deficiency in proteins integral to HR (RAD51, RAD54, DSS1 and RPA1), Fanconi anemia (FANCA, FANCC and FANCD2) or DNA damage signaling (ATM, ATR, CHK1, CHK2 and NBS1) was demonstrated to be associated with sensitivity to PARP inhibitors.^18^ These results demonstrated that gene aberrations directly or indirectly affecting the DNA damage response (DDR) or DNA repair determine the sensitivity of the tumour to PARP inhibitors. However, the pathways involved are complex and interacting.

Many studies aim to expand the utility of PARP inhibitors to BRCA‐proficient tumours by combining PARP inhibitors with other surrogates to induce “synthetic lethality” in these tumours. The aforementioned gene aberrations that induce HR pathway deficiency can be exploited using small‐molecule inhibitors or RNA interference. The phosphoinositide 3‐kinase (PI3K) pathway is the most frequently altered pathway in human tumours and regulates a wide range of cancer cell processes, including cell cycle progression, survival, metabolism, motility and genomic instability.^61^ PI3K inhibitors have shown significant anti‐tumour activities in ovarian cancers both in vitro and in vivo.^62^ The PI3K inhibitor BKM120 was shown to impair DNA repair by HR and sensitize breast cancer cells to PARP inhibition regardless of the BRCA mutation status.^63^, ^64^ Moreover, this same PI3K inhibitor was demonstrated to decrease PI3K/AKT/mTOR signaling activity, impair DNA repair by HR and sensitize ovarian cancer cells to PARP inhibition regardless of the status of the PIK3CA or BRCA genes. In addition, BRCA downregulation was observed to be a potential biomarker for an effective response to the proposed combination treatment.^65^, ^66^ Cyclin‐dependent kinase (CDK)1 is a core component of the cell cycle and promotes cell cycle progression. CDK1 phosphorylates BRCA1, which is necessary for BRCA1 to form foci at sites of DNA damage. CDK1 inhibition impairs BRCA1 function, compromises the cellular capacity to repair DNA by HR and sensitizes cells to PARP inhibitors both in vitro and in vivo.^67^ CDK12 is one of the 9 significantly mutated genes in HGSOC and regulates the transcription of BRCA1 and several other DNA repair genes.^36^ In HGSOCs, CDK12 mutations impaired CDK12 kinase activity, reduced HR gene (including BRCA1, ATR, FANCI and FANCD2) levels, and decreased RAD51 foci formation, thus disrupting HR repair and predicting sensitivity to PARP inhibitors.^68^ A genome‐wide synthetic lethality screen for candidate PARP inhibitor sensitivity genes identified CDK12 deficiency as a clinically relevant biomarker for PARP inhibitor sensitivity.^69^ The phase I study of an oral PI3K inhibitor (BKM120 or BYL719) combined with olaparib in patients with recurrent triple‐negative breast cancer or HGSOC (NCT 01623349); the phase Ib study of olaparib combined with either an oral mTORC1/2 inhibitor (AZD2014) or an AKT inhibitor (AZD5363) in patients with recurrent endometrial, triple‐negative breast, ovarian, primary peritoneal or fallopian tube cancer (NCT 02208375); and the phase I study of veliparib combined with a CDK inhibitor (dinaciclib) in patients with advanced solid tumours (NCT 01434316) are ongoing.

Vascular endothelial growth factor receptor 3 (VEGFR3) inhibition in ovarian cancer cells led to decreased levels of BRCA1/2 and restored chemosensitivity in resistant cell lines with wild‐type BRCA2.^70^ In a randomized phase II trial (NCT 01116648), the combination of olaparib with cediranib (a highly potent inhibitor of VEGFR 1‐3) significantly improved the survival of patients with BRCA wild‐type, platinum‐sensitive recurrent high‐grade ovarian cancer to a level equivalent to that of their BRCA mutant counterparts, thus overcoming the requirement for BRCA mutations in mediating PARP inhibitor sensitivity.^71^ Histone deacetylase (HDAC) enzymes are important for the repair of DNA DSBs by HR. The inhibition of HDAC enzymes led to a significant reduction in the transcription of HR genes, including BRCA1 and RAD51, and sensitized HR‐proficient ovarian cancer cells to PARP inhibition both in vitro and in vivo.^72^, ^73^ Furthermore, combining PARP inhibition with metformin enhanced the anti‐tumour effects of PARP inhibitors in both BRCA‐deficient and BRCA‐proficient ovarian cancer cells.^74^ DNA methylation plays an essential role in regulating normal biological processes as well as carcinogenesis. DNA methyltransferase 1 (DNMT1) belongs to a family of enzymes responsible for maintaining cellular DNA methylation patterns. The combination of a DNMT inhibitor with a PARP inhibitor impaired the BRCA‐mediated DDR and sensitized PARP inhibitor‐resistant ovarian cancer to PARP inhibitor therapy regardless of BRCA status.^75^

### 2.3 Genomic scar analysis of “BRCA‐like” tumours

The gain or loss of large chromosomal regions or even whole chromosomes is termed genomic scarring and can be observed as copy number variations resulting from the failure of DNA damage repair.^76^ Genomic scar analysis is based on DNA repair pathways rather than on DNA repair genes. Since the mutational analysis of all the genes associated with HR pathways is increasingly complex,^46^ genomic scar analysis is believed to be more efficient in identifying “BRCA‐like” tumours and has thus been developed to discriminate between HR‐proficient and HR‐deficient tumours.^46^, ^77^, ^78^ Genomic scar analysis with methods such as the “MyChoice” test from Myriad Genetics and the “FoundationFocus” test from Foundation Medicine has been intensively evaluated in clinical trials of PARP inhibitors.

The MyChoice HRD assay assesses BRCA1/2 sequences and genomic scarring and calculates an HRD score comprising three biomarkers: loss of heterozygosity (LOH) (>15 Mb but shorter than the whole chromosome), telomeric allelic imbalance (TAI) (allelic imbalance not crossing the centromere but extending to the subtelomere) and large‐scale state transitions (LSTs) (chromosomal breaks between adjacent regions of >10 Mb after filtering variations of ≤3 Mb).^79^, ^80^ Tumours were scored on a scale of 0–100 with a cut‐off score of 42. Any tumour that scored ≥42 or had a deleterious or suspected deleterious BRCA1/2 mutation was considered to have defective HR repair; tumours scoring <42 were considered to have functional HR repair.^12^ These markers reflect the degree of tumour genomic instability and are highly associated with defects in DNA repair pathway genes in ovarian cancers. For example, LOH is highly correlated with defects in BRCA1/2, PTEN, FANCM and RAD51C.^81^ A high TAI score indicates DNA repair defects in BRCA1/2 wild‐type serous ovarian cancers.^82^ All BRCA1/2‐mutated tumours had high LST scores. High LST scores are thought to be more accurate for indicating HRD than BRCA mutation for indicating HRD.^83^

Three clinical trials aimed to investigate the potential role of the aforementioned biomarkers in predicting benefits from PARP inhibitors. The phase III NOVA trial, which prospectively assessed the myChoice HRD assay in the maintenance setting following platinum‐based chemotherapy, aimed to broaden the efficacy population to those who are HRD‐positive as determined by a combination of the three markers LOH, TAI and LST. In 174 tumour samples, the myChoice HRD assay identified 100% (68/68) of germline BRCA mutant tumours and 57% (61/106) of germline BRCA wild‐type tumours with HRD that would benefit from niraparib therapy. However, this assay did not have sufficient precision to deselect patients who would not benefit from niraparib, since a statistically significant PFS rate increase was demonstrated also in the HRD‐negative group.^12^, ^84^ The phase II ARIEL2 trial was aimed to assess the ability of tumour genomic LOH to predict the response to rucaparib in the treatment setting. Tumours scoring above the LOH cut‐off of 14% (LOH‐high) with the FoundationFocus assay were deemed HRD‐positive. In this trial, 80% of BRCA mutant tumours; 29% of BRCA wild‐type, high‐LOH tumours; and 10% of BRCA wild‐type, low‐LOH tumours responded to rucaparib. The phase III ARIEL3 trial, in which the LOH cut‐off was elevated to 16%, assessed the ability of tumour genomic LOH to predict the response to rucaparib in the maintenance setting following platinum‐based chemotherapy. The tumour genomic LOH test was not sufficiently precise to deselect patients who would not benefit from rucaparib; more than 30% of patients with BRCA wild‐type, low‐LOH tumours in the rucaparib group achieved clinical benefit from rucaparib for more than 1 year, but less than 5% in the placebo group experienced this benefit. These results demonstrated that these tests can be used to identify patients with BRCA wild‐type, platinum‐sensitive ovarian cancers who might benefit from PARP inhibitors. However, a negative result on these tests is not sufficiently precise to exclude a clinical benefit from PARP inhibitors among BRCA wild‐type ovarian cancers in either the treatment or maintenance setting.^11^, ^12^, ^85^ However, more clinical trials are warranted to further investigate the role of these HRD biomarkers in predicting benefits from PARP inhibitors.

## 3 MECHANISMS UNDERLYING PARP INHIBITOR RESISTANCE

As increasing numbers of PARP inhibitors are used in the clinic, and the potential candidates have usually received many lines of chemotherapy, the investigation of the resistance mechanism is urgent to inform the administration of PARP inhibitors. The most common acquired mechanism of resistance to PARP inhibition is the restoration of BRCA1 or BRCA2 protein functionality by secondary mutations. In addition, this mechanism is shared by platinum resistance and PARP inhibitor resistance. PARP inhibitor‐resistant human pancreatic cancer cell lines were found to express new BRCA2 isoforms by an intragenic deletion of the c.6174delT frameshift mutation, which restored the open reading frame (ORF) of the BRCA2 gene and thus the ability to repair DSBs by HR repair.^86^, ^87^ The in vitro selection of a BRCA2‐mutated ovarian cancer cell line, which was sensitive to both platinum and PARP inhibition, by a cisplatin/PARP inhibitor combination led to the recovery of BRCA2 function induced by secondary BRCA2 mutation. Ovarian cancer cells extracted from ascites of platinum‐resistant relapsed ovarian cancers were found to harbour secondary BRCA2 mutations and to be BRCA2‐proficient. The depletion of BRCA2 resensitized these cells to the cisplatin/PARP inhibitor combination.^88^ In addition, secondary mutations in RAD51C or RAD51D were associated with resistance to PARP inhibition. Core HR pathway genes were sequenced in 12 pairs of pre‐treatment and post‐progression tumour biopsy samples from patients in ARIEL2 Part 1, a phase II study of the PARP inhibitor rucaparib as treatment for platinum‐sensitive, relapsed ovarian cancers. Functional mutations in BRCA1, RAD51C or RAD51D were identified in 6 of the 12 pre‐treatment biopsies. One or more secondary mutations in BRCA1, RAD51C or RAD51D that restored the ORF of these genes was identified in five of six paired post‐progression biopsies. These secondary mutations leading to resistance to the PARP inhibitor rucaparib were confirmed in both PDX models and in vitro assays.^89^

The frequency of the secondary mutation event was investigated in both platinum‐sensitive and platinum‐resistant BRCA‐mutated ovarian cancers. One (25.0%) in four primary platinum‐resistant versus zero (0%) in 56 primary platinum‐sensitive, BRCA‐mutated ovarian cancers, as well as 12 (46.2%) platinum‐resistant versus one (5.3%) platinum‐sensitive recurrent BRCA‐mutated ovarian cancers, exhibited secondary mutations in BRCA1/2 that restored the ORF of the gene. Six platinum‐resistant BRCA‐mutated ovarian cancers were treated with olaparib; two exhibited primary resistance to olaparib; two, a partial response; and two, a complete response. The two tumours with primary resistance to olaparib and one tumour exhibiting a partial response were found to harbour secondary mutations of BRCA2 that restored the ORF of the gene. The two tumours exhibiting a complete response and one tumour exhibiting a partial response did not exhibit secondary mutations.^23^ Sequencing of two samples of olaparib‐resistant tumours from clinical trials identified secondary BRCA2 mutations that restored the ORF of the gene.^90^ However, in another study, deep sequencing of six olaparib‐resistant tumours indicated no evidence of secondary BRCA mutation.^33^

BRCA1/2‐mutated ovarian cancers harbouring secondary mutations and exhibiting progression following platinum treatment may be resistant to both platinum and PARP inhibitors. However, platinum‐resistant, BRCA1/2‐mutated ovarian cancers without secondary mutations are likely to be sensitive to PARP inhibitors. Thus, testing secondary mutations may inform treatment options for ovarian cancer patients.^87^ For example, 6‐thioguanine has been demonstrated to kill cells and tumours that have gained resistance to PARP inhibitors via the genetic reversion of the BRCA2 gene.^32^, ^91^

Homologous recombination and NHEJ are the two pathways responsible for DSB repair. Error‐prone NHEJ induces genomic instability and can cause deleterious damage to HRD cells. KU70, KU80 and DNA‐PKcs, which are crucial proteins in the NHEJ pathway, can bind poly (ADP‐ribose) polymers generated by PARP enzymes and limit NHEJ pathway activity. Thus, the inhibition of PARP enzymes by PARP inhibitors would decrease the combined activity of these NHEJ proteins and poly (ADP‐ribose) polymers, thus promoting NHEJ and genomic instability.^27^ Functional studies demonstrated that NHEJ deficiency was independent of HR proficiency and was associated with resistance to PARP inhibitors in ex vivo primary cultures. NHEJ‐proficient/HR‐deficient ovarian cancer cells were more sensitive to rucaparib, than NHEJ‐proficient/HR‐proficient cells (*p* = 0.034), NHEJ‐deficient/HR‐proficient cells (*p* = 0.0002), and NHEJ‐deficient/HR‐deficient cells (*p* = 0.0045).^92^ The inhibition of DNA‐PK induced both the resistance to rucaparib and the recovery of HR function in a BRCA1‐defective cell line.^92^ The loss of Artemis, another critical factor in NHEJ, was found to be associated with resistance to niraparib in an HGSOC PDX model.^19^ Thus, the synthetic lethality between PARP inhibition and HR deficiency requires the concomitant competence of the NHEJ pathway.

p53‐binding protein 1 (53BP1) is a critical mediator of the DDR, which regulates the balance between the high‐fidelity HR pathway and the more error‐prone NHEJ pathway. 53BP1 loss promotes the end resection of DNA DSBs in the absence of BRCA1, resulting in RAD51 recruitment and HR restoration.^31^, ^32^, ^93^ Compared with the parental BRCA1‐deficient breast cancer cells, breast cancer cells with dual 53BP1 and BRCA1 deficiency displayed a reduced sensitivity of up to 36‐fold to PARP inhibitors. Seventy‐four percent of BRCA1‐deficient xenografts versus 7% of xenografts with dual 53BP1 and BRCA1 deficiency exhibited sensitivity to the PARP inhibitor simmiparib. Furthermore, the restoration of 53BP1 expression in the dual‐deficient cells restored the sensitivity to PARP inhibition.^94^ The loss of 53BP1 was demonstrated in 3 of 11 mice with BRCA1‐deficient mammary gland tumours, which were initially sensitive to PARP inhibitors but subsequently developed resistance,^29^ and twenty percent of PARP inhibitor‐resistant PDXs with germline BRCA1 mutations exhibited a loss of 53BP1.^95^ REV7, which plays a crucial function as a downstream effector of 53BP1 in coordinating pathological DSB repair pathway choices in BRCA1‐deficient cells, is recruited to DSBs and blocks DSB resection in order to promote NHEJ. The loss of REV7 in mouse and human cell lines re‐establishes the end resection of DSBs in BRCA1‐deficient cells, thus leading to HR restoration and PARP inhibitor resistance, which can be reversed by ATM inhibitors.^96^ Heat shock protein 90 (HSP 90) stabilized the C‐terminal (BRCT) domain of the mutant BRCA1 protein, which interacted with the PALB2‐BRCA2‐RAD51 complex and conferred PARP inhibitor resistance. Treating resistant cells with an HSP 90 inhibitor reduced mutant BRCA1 protein levels and restored sensitivity to PARP inhibition.^97^, ^98^

The increased expression of the ATP‐dependent efflux pump ABCB1 (MDR1), which encodes the membrane drug efflux transporter P‐glycoprotein, might readily export PARP inhibitors from tumour cells and lead to resistance to the PARP inhibitors olaparib or rucaparib but not to veliparib or AZD2461, both of which are poor P‐glycoprotein substrates.^29^, ^99^, ^100^ However, this resistance can be reverted using the ABCB1 inhibitors verapamil, elacridar and tariquidar.^101^

PARP1 is a nuclear enzyme that is activated by DNA damage and plays a critical role in BER. The inhibition of PARP1 is not equivalent of PARP1 deletion and the mechanisms of action for PARP inhibitors are based on both the catalytic inhibition of PARP1 enzyme and the trapping of PARP1‐DNA complexes. In vitro studies have demonstrated that the PARP inhibitor olapairb has no effect on cells with complete absence of the PARP1 enzyme. In the presence of PARP inhibitors, dysfunctional PARP1 enzymes bind DNA and form PARP1‐DNA complexes and PARP inhibitors promote trapping of these PARP1‐DNA complexes that are toxic to the cell.^102^ A certain amount of functional PARP1 is critical to tumour responses to PARP inhibitors because PARP1 is required both as a substrate for PARP1 trapping and for the cytotoxicity of PARP inhibitors. The deletion of PARP1 has been demonstrated to cause resistance to all PARP inhibitors in ovarian cancer cell lines in vitro.^103^ The PARP1 expression level is positively correlated with PARP inhibitor sensitivity.^104^ For example, an acquired low expression level of PARP1 is a potential cause of resistance to PARP inhibitors in PDX models.^103^ Furthermore, cells with PARP1 mutations were 100‐fold more resistant to PARP inhibitors than were cells with wild‐type PARP1.^105^ Mutations both within and outside the PARP1 DNA‐binding domains alter PARP1 trapping and induce PARP inhibitor resistance.^106^ Cancer cells may up‐regulate the HR repair pathway to compensate for the loss of BER as a result of PARP1 inhibition, and the HR and BER pathways interact to regulate cancer cell viability via decreased PARP1 and increased RAD51 expression levels. This mechanism may explain the concomitant RAD51 foci formation and PARP inhibitor resistance in both PDX models and patient‐derived samples harbouring germline BRCA mutations.^107^ In addition, this resistance was observed to be reverted upon combination treatment with an ATM inhibitor in PDX models.^95^

## 4 CONCLUSIONS

Homologous recombination deficiency, which can be investigated via mutational analysis of HRD gene panels, genomic scar analysis and functional assays, remains a strong predictor of clinical benefit from PARP inhibitors. However, the HRD biomarker apparently cannot efficiently identify the subgroup of patients with wild‐type BRCA that will achieve a significant increase in PFS with PARP inhibitor treatment. This inability may result from other HRD mechanisms not detected by current assays or from alternative explanations for PARP inhibitor sensitivity. In addition, numerous combination treatment strategies can induce HR pathway deficiency. Notably, the response to platinum‐based chemotherapy remains a strong predictor of the response to PARP inhibitor therapy, especially for BRCA‐proficient ovarian cancer. Mechanisms of resistance to PARP inhibition include the development of secondary mutations, deficiencies in the NHEJ pathway, the loss of 53BP1 expression, increases in drug export and decreases in PARP1 expression. The importance of these mechanisms of PARP inhibitor resistance in clinical settings and the identification of strategies to overcome this resistance warrant further investigation.

## CONFLICTS OF INTEREST

The authors declare that they have no conflicts of interest.
